# Linc-RAM promotes muscle cell differentiation via regulating glycogen phosphorylase activity

**DOI:** 10.1186/s13619-022-00109-8

**Published:** 2022-03-07

**Authors:** Lili Zhai, Xin Wan, Rimao Wu, Xiaohua Yu, Hu Li, Ran Zhong, Dahai Zhu, Yong Zhang

**Affiliations:** 1 The State Key Laboratory of Medical Molecular Biology, Institute of Basic Medical Sciences, Chinese Academy of Medical Sciences and School of Basic Medicine, Peking Union Medical College, Beijing, 100005 China; 2 Present address: NCPC New Drug Research and Development Co., Ltd., State Key Laboratory of Antibody Research & Development, Shijiazhuang, 052165 China; 3 The Max-Planck Center for Tissue Stem Cell Research and Regenerative Medicine, Bioland Laboratory (Guangzhou Regenerative Medicine and Health Guangdong Laboratory), Guangzhou, 510005 China

**Keywords:** Muscle cell differentiation, Long non-coding RNAs, Cytoplasm, Linc-RAM, Glycogen phosphorylase

## Abstract

Long non-coding RNAs (lncRNAs) are important regulators of diverse biological processes, especially skeletal muscle cell differentiation. Most of the lncRNAs identified to date are localized in the nucleus and play regulatory roles in gene expression. The cytoplasmic lncRNAs are less well understood. We previously identified a long intergenic non-coding RNA (linc-RNA) activator of myogenesis (Linc-RAM) that directly binds MyoD in the nucleus to enhance muscle cell differentiation. Here, we report that a substantial fraction of Linc-RAM is localized in the cytoplasm of muscle cells. To explore the molecular functions of cytoplasmic Linc-RAM, we sought to identify Linc-RAM-binding proteins. We report here that Linc-RAM physically interacts with glycogen phosphorylase (PYGM) in the cytoplasm. Knockdown of *PYGM* significantly attenuates the function of Linc-RAM in promoting muscle cell differentiation. Loss-of-function and gain-of function assays demonstrated that PYGM enhances muscle cell differentiation in an enzymatic activity-dependent manner. Finally, we show that the interaction between Linc-RAM and PYGM positively regulates the enzymatic activity of PYGM in muscle cells. Collectively, our findings unveil a molecular mechanism through which cytoplasmic Linc-RAM contributes to muscle cell differentiation by regulating PYGM activity. Our findings establish that there is crosstalk between lncRNAs and cellular metabolism during myogenic cell differentiation.

**Supplementary Information:**

The online version contains supplementary material available at 10.1186/s13619-022-00109-8.

## Background

RNA deep sequencing and functional genomics analyses have demonstrated that a significant number of noncoding RNAs (ncRNAs) are encoded in the human genome and those of other model organisms (Harrow et al., 2012). The long-noncoding RNAs (lncRNAs) comprise a subgroup of ncRNAs that are > 200 nt in length. A recent study employing an lncRNA-knockout (KO) mouse approach indicated that lncRNAs are functionally relevant in regulating cell differentiation and development: Individual KO of 18 different lncRNAs led to a variety of developmental defects affecting diverse organs, including the lungs, gastrointestinal tract, and heart (Sauvageau et al., 2013). An increasing number of lncRNAs have been reported to have profound functions in regulating various aspects of cellular biology. Specific mechanisms have been clearly defined for a few well-studied lncRNAs, yielding new insights into the functions of these RNAs.

The wide-ranging effects of different lncRNAs are closely linked to their interaction with RNA-binding proteins (RBPs) in the cytoplasm or nucleic acids in the nucleus. The many nuclear lncRNAs have been extensively studied, and such work has revealed that they function in protein complexes that play structural and regulatory roles to enable gene organization and control transcription (Noh et al., 2018). The cytoplasmic lncRNAs are less well understood, but accumulating evidence indicates that they also form complexes with diverse structural and regulatory proteins. One of the first functional mechanisms attributed to a cytoplasmic lncRNA was that of acting as an miRNA sponge during muscle cell differentiation and muscular disease. The lncRNA, Linc-MD1, has been shown to control muscle cell differentiation in both mouse and human myoblasts through its ability to bind miR-133 and miR-135, thereby alleviating repression of mastermind-like transcriptional coactivator–1 (*MAML1*) and myocyte enhance factor 2C (*MEF2C*), respectively (Cesana et al., 2011). Other muscle-relevant competing endogenous RNAs (ceRNAs) have also been identified, including the lncRNAs, H19 (Kallen et al., 2013), cardiac hypertrophy related factor (CHRF) (Wang et al., 2014), and adenocarcinoma transcript (MALAT1) (Han et al., 2015). Several lncRNAs that exert their functions by controlling mRNA stability and translation have also been linked to myogenesis. Staufen1-mediated mRNA decay (SMD) of mRNA has been shown to occur in muscle cells via intermolecular base pairing between short interspersed element (SINE)-containing lncRNAs (m1/2-sbsRNAs) and SINE-containing mRNA 3’UTRs (Wang et al., 2013). The cytoplasmic lncRNAs can also serve as “decoys” to regulate the availability of RNA-binding proteins in muscle cells. The LncMyoD controls cell-cycle exit during myoblast differentiation by binding IGF2-mRNA-binding protein 2 (IMP2) to reduce IMP2-mediated mRNA translation (Gong et al., 2015). Although great progress has been made in elucidating the functions of cytoplasmic lncRNAs, further in-depth investigation is needed to clarify the underlying mechanisms.

We previously identified and characterized a long intergenic non-coding RNA (linc-RNA) activator of myogenesis (Linc-RAM) that promotes myogenic cell differentiation by facilitating the assembly of the MyoD–Baf60c–Brg1 complex on the regulatory elements of target genes in the nucleus (Yu et al., 2017). Here, we report that Linc-RAM also distributes in the cytoplasm of muscle cells. Cytoplasmic Linc-RAM binds to glycogen phosphorylase (PYGM) and regulates its enzymatic activity, which is indispensable for muscle cell differentiation. Our findings uncover an RNA regulator of glycogenolysis that links lncRNAs and cellular metabolism during muscle cell differentiation.

## Results

### Linc-RAM directly interacts with glycogen phosphorylase (PYGM) in the cytoplasm

**Fig. 1:**
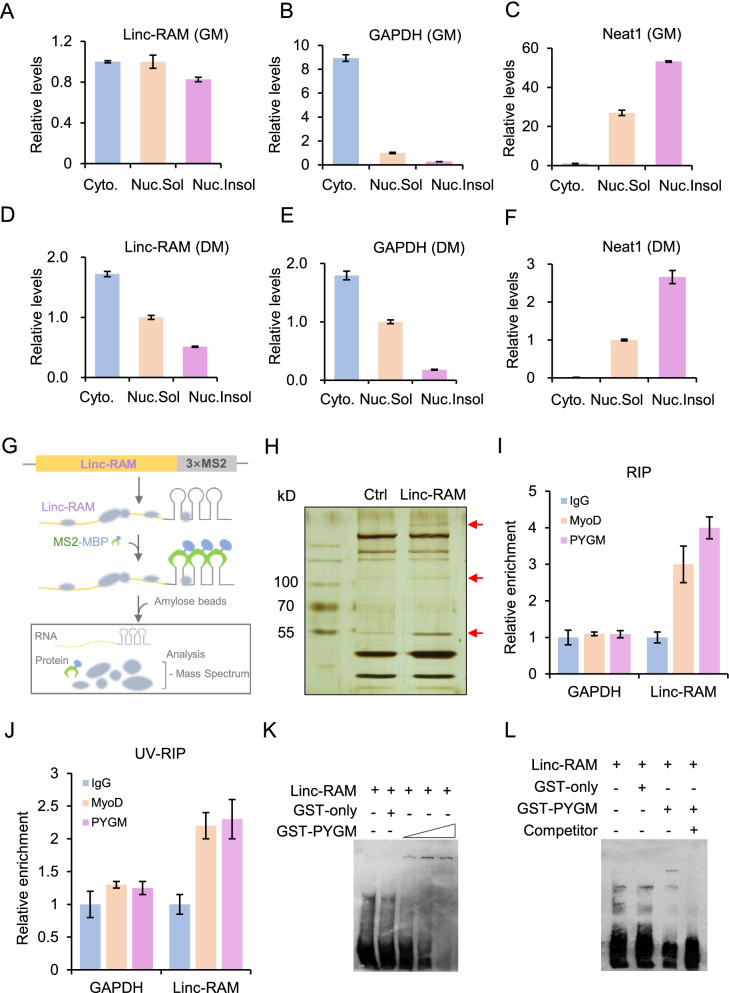
Linc-RAM directly interacts with PYGM in the cytoplasm. **A-F** Linc-RAM in cytoplasmic (Cyto), nuclear-soluble (Nuc.Sol), and nuclear-insoluble (Nuc.Insol) fractions of C2C12 cells cultured in growth medium (**A-C**) and differentiation medium for 24 h (**D-F**), as determined by RT–qPCR. The *GAPDH* mRNA was used as a marker for the cytoplasmic fraction. Neat1 (nuclear paraspeckle assembly transcript 1) was used as a marker for the nuclear fraction. The data are representative of three independent experiments. **G** Schematic diagram showing the strategy applied to identify Linc-RAM-binding proteins using MS2-MBP-mediated RNA pull down. Three bacteriophage MS2 coat protein-binding sites (3 × MS2 hairpins) were fused to the 3′-end of Linc-RAM (Linc-RAM-3 × MS2). MS2-MBP represents a fusion protein comprising MS2 coat protein and maltose-binding protein. **H** A representative silver-stained SDS-PAGE gel showing the bands that differed (red arrow) between Linc-RAM-3 × MS2 (Linc-RAM) and the 3 × MS2 control (Ctrl). The differential bands were individually extracted and subjected to mass spectrometry (MS) analysis. **I**, **J** RNA immunoprecipitation (RIP) analysis to validate the physical interaction between Linc-RAM and PYGM in C2C12 cells cultured in differentiation medium for 24 h. Native (**I**) or UV-crosslinked (**J**) C2C12 cells differentiated for 24 h were immunoprecipitated using anti-PYGM, anti-MyoD, and IgG antibodies. Linc-RAM in immunoprecipitates was examined by RT–qPCR. *GAPDH* served as a negative control. MyoD antibodies served as a positive control, as we previously reported that Linc-RAM binds MyoD (Yu et al., 2017). **K** Representative RNA electrophoretic mobility shift assay (EMSA) results obtained using biotin-labeled Linc-RAM and different doses of recombinant GST-PYGM fusion protein (50 ng, 100 ng, 200 ng). The biotin-labeled Linc-RAM and recombinant PYGM protein complex were resolved on a 5% native polyacrylamide gel and subsequently the Linc-RAM/PYGM complex was detected by HRP-Streptavidin. Recombinant GST protein (GST-only) served as a negative control. **L** Representative RNA EMSA results obtained using biotin-labeled Linc-RAM and recombinant GST-PYGM fusion protein (200 ng). As competitors, non-labeled Linc-RAM probes were added to confirm the binding specificity. The presented values reflect the means ± SE obtained from three independent experiments

We previously reported that the lncRNA, Linc-RAM, enhances myogenic differentiation by interacting with MyoD in the nucleus (Yu et al., 2017). Here, we found that Linc-RAM was also distributed in the cytoplasm of both proliferating and differentiated muscle cells (Fig. 1A-F). In addition, it was more in the cytoplasm than that in the nuclear fractions during muscle cell differentiation (Fig. 1D). To unveil the molecular functions of cytoplasmic Linc-RAM in regulating early differentiation of muscle cells, we identified Linc-RAM-binding proteins using an MS2-MBP system in which MS2-tagged RNA was pulled down with a fusion protein comprising MS2 coat protein and maltose-binding protein (MS2-MBP) (Zhou & Reed, 2003) (Fig. 1G). C2C12 cells (a muscle stem cell-derived cell line) were transfected with plasmids expressing MS2-tagged Linc-RAM (Linc-RAM-3 × MS2) and differentiation was induced for 24 h. The empty vector solely expressing 3 × MS2 RNA served as a control. Cytoplasmic fractions of the differentiated cells were incubated with purified recombinant MS2-MBP fusion protein, and the ternary RNA/protein complex (endogenous Linc-RAM-binding proteins, Linc-RAM-3 × MS2, and MS2-MBP) was pulled down with amylose beads. The proteins that exhibited differential binding compared to the 3 × MS2 control were collected and subjected to mass spectrum (MS) analysis (Fig. 1H). Several candidates were obtained from the MS data (Supplementary Table 1). After matching the molecular weight and subcellular localization, we selected glycogen phosphorylase (PYGM) for further validation and functional analysis.

Firstly, we validated whether endogenous Linc-RAM physically interacts with PYGM in muscle cells by performing native RNA immunoprecipitation (RIP) and UV-crosslinked RIP (UV-RIP) with an anti-PYGM antibody. The immunoprecipitated RNAs were examined by reverse transcription followed by quantitative PCR (RT–qPCR) using primers specific for Linc-RAM. As shown in Fig. 1I and J, the Linc-RAM transcript was enriched by the anti-PYGM antibody but not the anti-IgG control, indicating that Linc-RAM physically associates with PYGM in muscle cells. As a positive control, the anti-MyoD antibody successfully pulled down the Linc-RAM transcript. The glyceraldehyde-3-dehydrogenase (*GAPDH*) transcript, which was used as a negative control, was not detected in the immunoprecipitated samples (Fig. 1I,J), confirming the specificity of the anti-PYGM antibody. Next, to further assess the direct interaction between Linc-RAM and PYGM, we performed electrophoretic mobility shift assays (EMSAs) followed by reconstitution experiments using in vitro-transcribed Linc-RAM and purified recombinant GST-PYGM fusion protein (Fig. S1). We found that Linc-RAM directly interacted with GST-PYGM, but not with GST alone (Fig. 1K,L), and this specific interaction was abolished by cold competitor probes (Fig. 1L). Taken together, our results demonstrate that Linc-RAM directly interacts with PYGM in the cytoplasm of muscle cells.

### PYGM promotes muscle cell differentiation

**Fig. 2:**
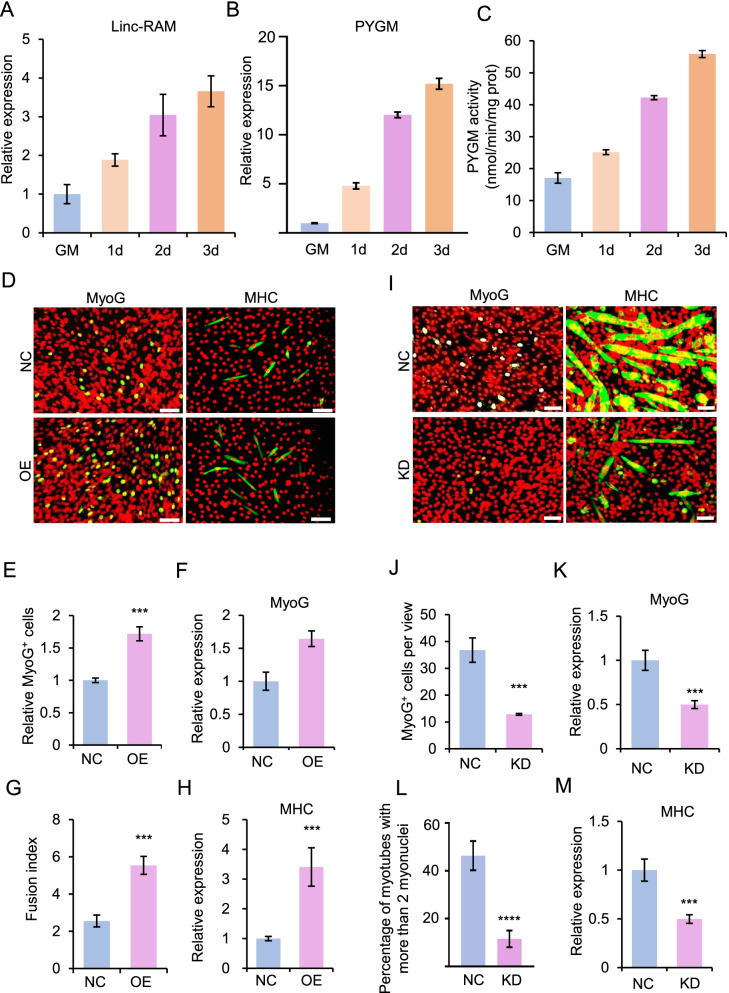
PYGM promotes C2C12 cell differentiation. **A** Relative expression of Linc-RAM in C2C12 cells cultured in growth medium (GM) or differentiation medium for 1 day (1d), 2 days (2d) or 3 days (3d), determined by real-time RT-qPCR. **B** Relative expression of *PYGM* in C2C12 cells described in **A**, determined by real-time RT-qPCR. **C** Enzymatic activity of PYGM in C2C12 cells described in (**A). D** Representative images of immunostaining for MyoG (green) or MHC (green) in C2C12 cells transfected with plasmids expressing PYGM (OE) and cultured in differentiation medium for 24 h (MyoG) or 48 h (MHC). Transfection with empty vector served as a negative control (NC). DAPI (pseudo-colored red) served to visualize nuclei. Scale bars, 100 μm. **E** Relative numbers of the MyoG-positive (MyoG^+^) cells described in (**D**). **F** Relative expression of *MyoG* in the cells described in (**D**), as determined by RT–qPCR. **G** Fusion index calculated in the cells described in (**D**). **H** Relative expression of *MHC* in the cells described in (**D**), as determined by RT–qPCR. **I** Representative images of immunostaining for MyoG (green) or MHC (green) in C2C12 cells transfected with siRNA against PYGM (KD) and cultured in differentiation medium for 24 h (MyoG) or 72 h (MHC). Transfection with scramble RNA served as a negative control (NC). DAPI (pseudo-colored red) served to visualize nuclei. Scale bars, 50 μm. **J** Numbers of MyoG-positive (MyoG^+^) cells per view described in (**I**). **H** Relative expression of *MyoG* in the cells described in (**I**), as determined by RT–qPCR. **L** Percentage of myotubes with more than 2 myonuclei calculated in the cells described in (**I**). **M** Relative expression of *MHC* in the cells described in (**I**), as determined by RT–qPCR. All images are representative of three independent experiments. Values presented represent the means ± SE obtained from three independent experiments. The statistical significance of the difference between two means was calculated with the Student’s t-test. *p < 0.05, **p < 0.01, ***p < 0.001

The observation that Linc-RAM directly binds PYGM prompted us to investigate the functional role of PYGM during muscle cell differentiation. To this end, we firstly examined expression pattern and enzymatic activity of PYGM during muscle cell differentiation. The C2C12 cells were differentiated for 1, 2 and 3 days, respectively. Expression of *PYGM* and Linc-RAM were measured by real-time RT-PCR. The enzymatic activity of PYGM during the same time points was analyzed. We found that RNA levels of *PYGM* and Linc-RAM were upregulated during muscle cell differentiation (Fig. 2A,B). Consistently, the enzymatic activity of PYGM was also gradually increased with the progression of cell differentiation (Fig. 2C). Together, our data suggest PYGM play roles in regulating muscle cell differentiation.

Next, we examined effects of PYGM overexpression on muscle cell differentiation. The C2C12 cells transiently overexpressing (OE) PYGM were cultured in differentiation medium (DM) for 24 h or 48 h. Immunostaining of DM conditioned for 24 h for the early myogenic differentiation marker, myogenin (MyoG), revealed that there were significantly more differentiating cells in PYGM OE cells than in control cells (Fig. 2D,E). The mRNA level of *MyoG* in PYGM OE cells was also significantly higher than that in control cells, as determined by RT-qPCR (Fig. 2F). The idea that PYGM promotes C2C12 cell differentiation was further supported by our analysis of the late-stage myogenic differentiation marker, myosin heavy chain (MHC) in DM conditioned for 48 h. PYGM significantly increased the number of MHC-positive cells (Fig. 2D), the fusion index (Fig. 2G), and the level of *MHC* mRNA (Fig. 2H) in PYGM OE cells compared to control cells.

**Fig. 3:**
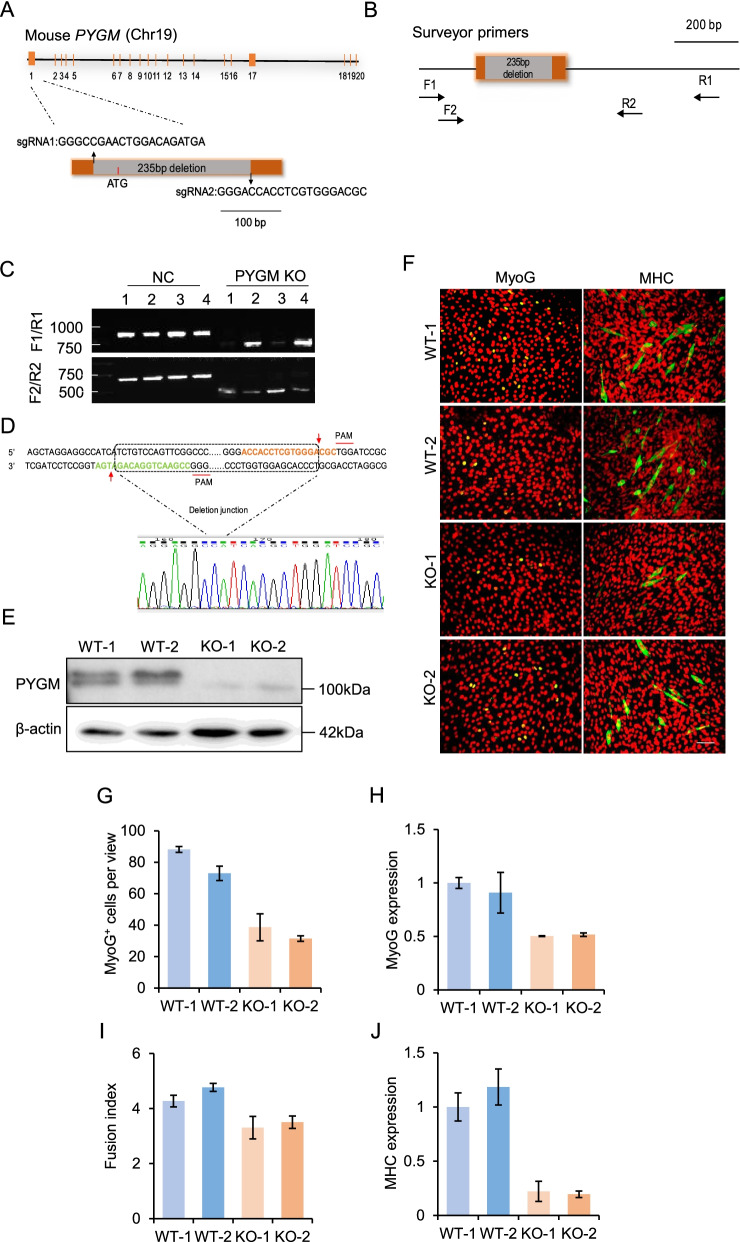
Loss of PYGM function delays C2C12 cell differentiation. **A** Two sgRNAs designed to target the first exon of *PYGM*. The resulting allele harbors a 235-bp deletion in exon 1. **B** Schematic illustration of the surveyor primers used to identify *PYGM*-knockout clones. **C** *PYGM*-knockout clones (1, 2, 3, 4) were validated by RT-PCR. Wild-type clones (1, 2, 3, 4) served as controls. **D** *PYGM*-knockout clone verified by DNA sequencing. **E** Western blotting analysis to verify *PYGM*-knockout clones (KO-1, KO-2). Wild type clones (WT-1, WT-2) served as positive controls. β-actin served as an equal-loading control. **F** Representative images of immunostaining for MyoG (green) or MHC (green) in *PYGM*-knockout (KO-1, KO-2) and wild-type (WT-1, WT-2) C2C12 cells cultured in differentiation medium for 24 h (MyoG) or 48 h (MHC). DAPI (pseudo-colored red) served to visualize nuclei. Scale bars, 100 μm. **G** Numbers of MyoG-positive (MyoG^+^) cells per view described in (**F**). **H** Relative expression of *MyoG* in the cells described in (**F**), as determined by RT–qPCR. **I** Fusion index calculated in the cells described in (**F**). **J** Relative expression of *MHC* in the cells described in (**F**), as determined by RT–qPCR. All images in the figure are representatives of three independent experiments. Values presented indicate the means ± SE obtained from three independent experiments

To further corroborate the effect of PYGM on C2C12 cell differentiation, we knocked down *PYGM* in C2C12 cells using siRNAs. Loss of PYGM resulted in a significant decrease in the number of MyoG-positive cells (Fig. 2I,J) and the level of *MyoG* mRNA (Fig. 2K). PYGM-depleted cells that had undergone differentiation for 72 h showed a remarkably reduced number of MHC-positive cells (Fig. 2I), a smaller percentage of myotubes with more than 2 myonuclei (Fig. 2L), and a decreased level of *MHC* mRNA compared with control cells (Fig. 2M). In addition, we generated *PYGM*-knockout C2C12 cells using a CRISPR/Cas9 strategy (Fig. 3A-D) and evaluated their differentiation capabilities (Fig. 3E-G). Compared to wild-type (WT) control cells harboring non-targeting sgRNAs*, PYGM*-knockout cells exhibited fewer MyoG-positive cells (Fig. 3F, G), a smaller fusion index (Fig. 3F, I), and lower mRNA levels of *MyoG* and *MHC* (Fig. 3H, G). Together, these results demonstrate that PYGM significantly potentiates muscle cell differentiation in vitro.

### Linc-RAM promotes muscle cell differentiation in a PYGM-dependent fashion

**Fig. 4:**
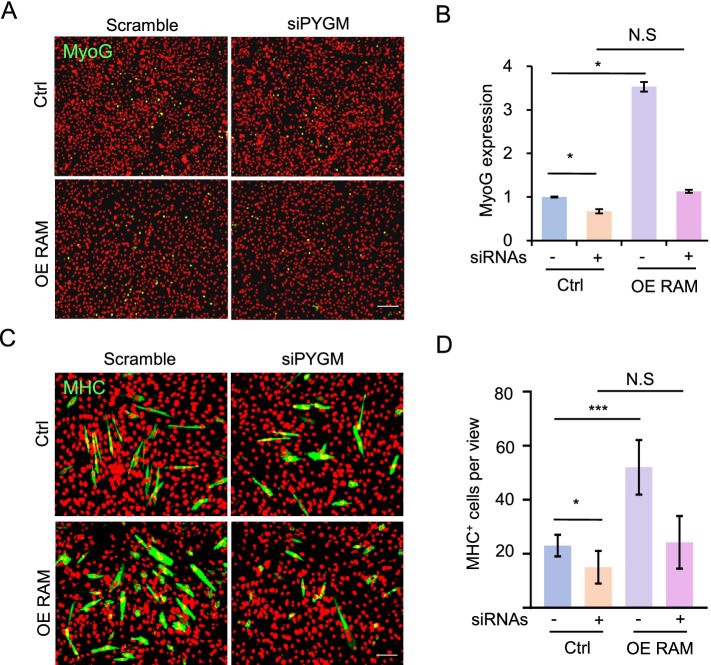
Linc-RAM enhances muscle cell differentiation in a PYGM-dependent manner. **A** Representative images of immunostaining for MyoG (green) in C2C12 cells subjected to overexpression of Linc-RAM (OE) with simultaneous siRNA knockdown of PYGM, followed by induction of differentiation for 24 h. Scramble RNA served as the siRNA control (Scramble). The empty vector served as the negative control (Ctrl) for overexpression of Linc-RAM. DAPI (pseudo-colored red) served to visualize nuclei. Scale bars, 200 μm. **B** Relative expression of *MyoG* in the cells described in (**A**), as determined by RT–qPCR. **C** Representative images of immunostaining for MHC (green) in C2C12 cells subjected to overexpression of Linc-RAM (OE) with simultaneous siRNA-mediated knockdown of PYGM, followed by induction of differentiation for 48 h. Scramble RNA served as the siRNA control (Scramble). The empty vector served as the negative control (NC) for overexpression of Linc-RAM. DAPI (pseudo-colored red) served to visualize nuclei. Scale bars, 100 μm. **D** Numbers of MHC-positive (MHC+) cells per view calculated in the cells described in (**C**). All images are representatives of three independent experiments. Values presented indicate the means ± SE obtained from three independent experiments, and were compared by two-way ANOVA. *p < 0.05, **p < 0.01. NS: statistically non-significant

We next asked whether PYGM is required for Linc-RAM function in regulating muscle cell differentiation. To do that, we knocked down *PYGM* using specific siRNAs in Linc-RAM-overexpressing (OE) cells. After differentiation was induced for 24 h or 48 h, the C2C12 cells were immunostained for the muscle cell differentiation markers, MyoG (24 h, Fig. 4A) or MHC (48 h, Fig. 4C), and the MyoG-positive cells (Fig. 4B) and fusion index (Fig. 4D) were calculated. Consistent with the previous report (Yu et al., 2017), we found that overexpression of Linc-RAM significantly enhanced muscle cell differentiation, as evidenced by increased proportions of MyoG- and MHC-positive cells (Fig. 4A-D). However, in *PYGM*-knockdown cells, Linc-RAM was unable to promote muscle cell differentiation (Fig. 4A-D), indicating that PYGM is required for the ability of Linc-RAM to confer its regulatory roles in muscle cells. Thus, Linc-RAM promotes muscle cell differentiation in a PYGM-dependent fashion.

### The enzymatic activity of PYGM is required for muscle cell differentiation

**Fig. 5:**
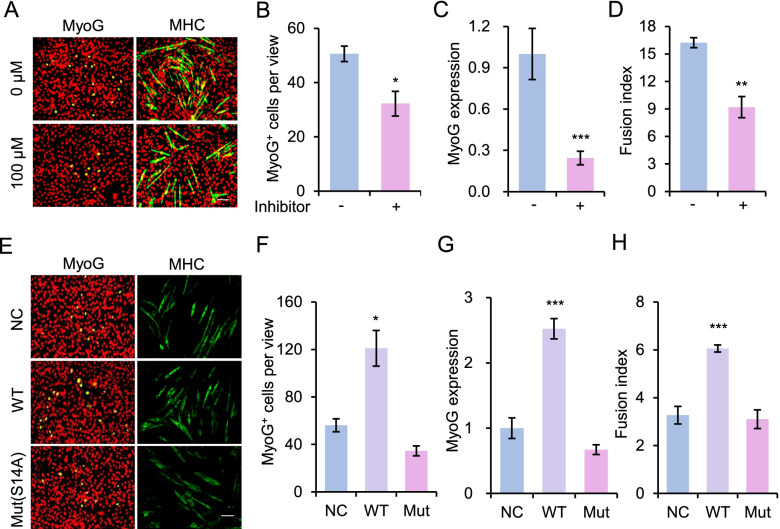
The enzymatic activity of PYGM is required for muscle cell differentiation. **A** Representative images of immunostaining for MyoG (green) or MHC (green) in C2C12 cells treated with 100 μM of PYGM activity inhibitor (C5H11NO3·HCl, Sigma, D1542) and cultured in differentiation medium for 24 h (MyoG) or 48 h (MHC). DAPI (pseudo-colored red) served to visualize nuclei. Scale bars, 100 μm. **B** Numbers of MyoG-positive (MyoG^+^) cells per view described in (**A**). **C** Relative expression of *MyoG* in the cells described in (**A**), as determined by RT–qPCR. **D** Fusion index calculated in the cells described in (**A**). **E** Representative images of immunostaining for MyoG (green) or MHC (green) on C2C12 cells transfected with plasmids expressing wild-type (WT) or mutant PYGM (S14A) and induced to differentiate for 24 h (MyoG) or 48 h (MHC). Transfection with empty vector served as the negative control (NC). DAPI (pseudo-colored red) served to visualize nuclei. Scale bars, 100 μm. **F** Numbers of MyoG-positive (MyoG^+^) cells per view described in (**E**). **G** Relative expression of *MyoG* in the cells described in (**E**), as determined by RT–qPCR. **H** Fusion index calculated in the cells described in (**E**). All images are representatives of three independent experiments. Values presented represent the means ± SE obtained from three independent experiments. The statistical significance of the difference between two means was calculated with the Student’s t-test. *p < 0.05, **p < 0.01, ***p < 0.001

Given that PYGM is a key enzyme in glycogen metabolism and functions in regulating muscle cell differentiation, we asked whether the ability of PYGM to promote C2C12 cell differentiation depends on its enzymatic activity. C2C12 cells were treated with 100 μM of an agent (C5H11NO3·HCl, Sigma, D1542) that inhibits the enzymatic activity of PYGM for 24 h or 48 h in differentiation medium. The differentiation ability of cells was evaluated by immunostaining for the myogenic differentiation markers, MyoG and MHC, as shown in Fig. 5A. The MyoG-positive cell number (Fig. 5B) and fusion index (Fig. 5D) were significantly reduced in cells treated with the PYGM activity inhibitor. In line with this, the mRNA level of *MyoG* was much lower in cells treated with the PYGM activity inhibitor than in the DMSO-treated control (Fig. 5C). These data suggest that blocking the enzymatic activity of PYGM attenuates its ability to promote muscle cell differentiation and fusion.

Phosphorylation of PYGM at S14 is required for its enzymatic activity (Gaboriaud-Kolar & Skaltsounis, 2013). Thus, we further generated a mutant form (S14A) of PYGM (PYGM-Mut), with the wild-type form of PYGM (PYGM-WT) serving as a control. C2C12 cells were transfected with plasmids expressing PYGM-Mut or PYGM-WT, and differentiation was induced for 24 h or 48 h. We found that the MyoG-positive cell population (Fig. 5E, F), the mRNA level of *MyoG* (Fig. 5G), and the fusion index (Fig. 5E, H) were significantly higher in PYGM-WT cells than in the empty-vector control (NC). In contrast, these parameters did not significantly differ between PYGM-Mut and NC cells, indicating that the enzyme-dead form of PYGM lost its function in promoting muscle cell differentiation. Based on these results, we conclude that the enzymatic activity of PYGM is required for its regulatory roles in muscle cell differentiation.

### Linc-RAM regulates the enzymatic activity of PYGM

**Fig. 6:**
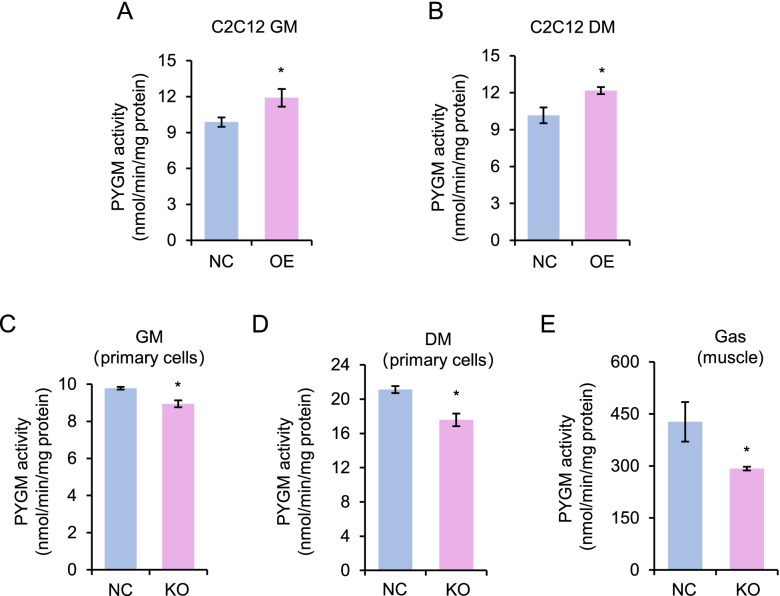
Linc-RAM regulates the enzymatic activity of PYGM. **A**, **B** Enzymatic activity of PYGM in C2C12 cells transfected with plasmids expressing Linc-RAM (OE) or empty vector as control (NC) and cultured in growth medium (**A**) or differentiation medium for 24 h (**B**). **C**, **D** Enzymatic activity of PYGM in primary myoblasts isolated from skeletal muscle of *Linc-RAM*-knockout mice (KO) or wild-type (NC) littermates and cultured in growth medium (**C**) or differentiation medium for 24 h (**D**). **E** Enzymatic activity of PYGM examined in skeletal muscle (gastrocnemius) from *Linc-RAM*-knockout mice (KO) or wild-type (NC) littermates. *n* = 3 per genotype. Values presented represent the means ± SE obtained from three independent experiments (**A-D**). The statistical significance of the difference between two means was calculated with the Student’s t-test. *p < 0.05

Based on the above findings, we speculated that cytoplasmic Linc-RAM physically interacts with PYGM and regulates its enzymatic activity to control muscle cell differentiation. To test the hypothesis, we examined the enzymatic activity of PYGM in Linc-RAM-overexpressing (RAM OE) C2C12 cells or *Linc-RAM*-knockout (RAM KO) primary myoblasts. Firstly, RAM OE C2C12 cells maintained in growth medium (GM) or differentiation culture for 24 h (DM) were collected and PYGM activities were measured. We found that PYGM activity was significantly higher in RAM OE cells than in control cells when cells were cultured in both GM and DM (Fig. 6A, B). Primary myoblasts isolated from *Linc-RAM* gene knockout mice (RAM KO) or wild-type (WT) littermates and cultured in growth medium or differentiation medium for 24 h were collected and PYGM activity was measured. We found that the PYGM activity was remarkably lower in primary myoblasts from RAM KO mice than from WT controls (Fig. 6C, D). Consistent with this observation, the PYGM activity was significantly reduced in skeletal muscle tissue (gastrocnemius) isolated from RAM KO mice compared to WT littermates (Fig. 6E). Together, these findings suggest that Linc-RAM interacts with PYGM and regulates its enzymatic activity in muscle cells.

## Discussion

The biological relevance of long non-coding RNAs in regulating development, cell differentiation, and growth has been documented. Most lncRNAs exert their functions in the nucleus to enable genome organization and control gene transcription (Sun et al., 2018). However, many lncRNAs, including H19 (Kallen et al., 2013), MALAT-1 (Han et al., 2015) and lnc-31/HG31 (Ballarino et al., 2015), are found in both the nucleus and cytoplasm. Cytoplasmic lncRNAs with various functions are increasingly being identified, but our understanding of their molecular mechanisms remains incomplete (Noh et al., 2018).

We previously demonstrated that Linc-RAM functions in the nucleus, where it regulates myogenic differentiation by directly binding MyoD to facilitate assembly of the epigenetic regulatory complex, MyoD–Baf60c–Brg1 (Yu et al., 2017). Interestingly, we herein report that Linc-RAM partially localizes in the cytoplasm and directly interacts with the key glycogen metabolism enzyme, PYGM, and that knockdown of *PYGM* significantly attenuates the function of Linc-RAM in promoting muscle cell differentiation. We thus describe a novel mechanism wherein cytoplasmic Linc-RAM controls muscle cell differentiation by regulating PYGM activity. Interestingly, recent study demonstrate *PYGM* is a target gene of MyoD during embryonic myogenesis (McQueen & Pownall, 2017), raising a possibility that nucleus-localized Linc-RAM concerts with MyoD to regulate *PYGM* gene transcription, and the cytosolic-localized Linc-RAM directly binds PYGM to regulate its enzymatic activity.

During muscle glycogenolysis, PYGM breaks down glycogen to glucose-1-phosphate (G1P); this is subsequently converted to glucose-6-phosphate (G6P), which can serve as a direct substrate for further catabolism via glycolysis to support ATP production and provide glucose for muscle contraction (Nielsen et al., 2011; Adeva-Andany et al., 2016). Despite this knowledge, however, it was unclear whether PYGM functions in early muscle cell differentiation. In the present study, we show that knockdown of *PYGM* with specific siRNAs or knockout of *PYGM* with the CRISPR/Cas9-system significantly delays C2C12 cell differentiation, whereas overexpression of PYGM enhances this parameter. It is interesting to consider how PYGM regulates muscle cell differentiation. Recent studies showed that glycogen-storing cells, such as those in muscle and brain tissues, can maintain intracellular glycogen reserves for cell-intrinsic metabolic requirements (Roach et al., 2012). Thwe et al. showed that dendritic cells (DCs) possess intracellular glycogen stores that fuel their activation-associated induction of glycolysis and their immune effector function. The authors uncovered a novel mechanism of metabolic regulation in DCs, wherein glucose- and glycogen-derived carbons preferentially contribute to distinct metabolic pathways (Thwe et al., 2017). In muscle cells, PYGM-mediated glycogenolysis might regulate cell differentiation via a mechanism similar to that described for DCs.

Our findings uncover a RNA regulator for glycogenolysis and link lncRNAs with cellular metabolism during muscle cell differentiation. Biochemically, the enzymatic activation of PYGM has been well documented (Gaboriaud-Kolar & Skaltsounis, 2013; Johnson, 1989; Barford & Johnson, 1989). Its tight regulations are achieved through seven major sites within each monomer (Baker et al., 2006; Wang, 1999; Newgard et al., 1989) as shown in Fig. S2. These include the catalytic site (C-site), glycogen site (G-site), nucleotide binding site (adenosine monophosphate (AMP)-site), phosphorylation site (P-site), ndole-site, inhibitor site, and 280 s’ loop. In this study, we demonstrate that Linc-RAM directly binds PYGM and regulates its enzymatic activity. Thus, it is intriguing to speculate on how Linc-RAM might regulate PYGM activity. One possibility might be that Linc-RAM facilitates the binding of AMP to PYGM or stimulates the displacement of the 280 s’ loop to allow the opening of the C-site (Buchbinder & Fletterick, 1996). In muscle cells, PYGM exists as an inactive tetramer and becomes activated as a dimer (Wang, 1999). Thus, another possibility is that Linc-RAM might function as a scaffold to mediate PYGM dimerization. Future work aimed at mapping the interaction domain(s) between Linc-RAM and PYGM would greatly help us understand how Linc-RAM regulates the enzymatic activity of PYGM.

## Conclusions

In summary, we herein unveil a novel mechanism by which cytoplasmic Linc-RAM regulates muscle cell differentiation. Cytoplasmic Linc-RAM binds PYGM and regulates its enzymatic activity, which is indispensable for muscle cell differentiation. Our findings uncover a RNA regulator for glycogenolysis, which links lncRNAs and cellular metabolism during muscle cell differentiation.

## Methods

### Mouse lines and animal care

All animal procedures were approved by the Animal Ethics Committee of Peking Union Medical College, Beijing (China). Mice were housed in an animal facility and given free access to water and standard rodent chow. The *Linc-RAM*-knockout mice in the C57BL/6j background were as previously described (Yu et al., 2017). Three-week-old *Linc-RAM*-knockout and wild-type littermate mice were used for the isolation of primary myoblasts.

### C2C12 cell culture and differentiation

Mouse C2C12 cells were cultured in growth medium consisting of Dulbecco’s modified Eagle’s medium (DMEM; Gibco, Life Technologies, Carlsbad, CA, USA) supplemented with 4.5 g/l glucose, 10% fetal bovine serum (FBS), and 1% penicillin/streptomycin at 37 °C in a 5% CO_2_ atmosphere. For differentiation of C2C12 myoblasts to myotubes, cells were transferred to DMEM containing 2% horse serum (HS) and 1% penicillin/streptomycin, and then cultured for the indicated durations. All cells were grown to ~ 80–90% confluence before the induction of differentiation.

### Isolation and culture of primary myoblasts

Hindlimb skeletal muscles were minced and digested with a mixture of type II collagenase and dispase B (Roche Applied Science, Basel, Switzerland). The obtained cells were filtered, centrifuged, and cultured in growth medium (F-10 Ham’s medium supplemented with 20% FBS, 4 ng/ml basic fibroblast growth factor, and 1% penicillin/streptomycin) on collagen-coated cell culture plates at 37 °C, 5% CO_2_. For differentiation, cells were transferred to differentiation medium (DM) containing 2% HS and then cultured for 24 h.

### RNA pull-down with MS2-MBP

To perform RNA pull-down assay, we firstly engineered a plasmid encoding 3 × MS2-tagged Linc-RAM. To do that, the Linc-RAM cDNA were generated by RT-PCR with total RNA from muscle cells. The PCR products of Linc-RAM cDNA were cloned into pCMV6-entry vector at upstream of 3 × MS2 sequences with Asf1 and MluI restriction enzymes. Subsequently, C2C12 cells were transfected with the plasmids encoding 3 × MS2-tagged Linc-RAM (pCMV6-entry-Linc-RAM-3 × MS2) and induced to differentiation for 24 h. The transfection with empty vector served as negative control (only encoding 3 × MS2). Subsequently, cytoplasmic fractions from 1 × 10^7^ cells were incubated with 4 μg of purified recombinant MS2-MBP protein in 0.1% NP-40 lysis buffer containing a protease inhibitor cocktail at 4 °C for 3 h. Then 100 μl of pretreated amylase magnetic beads (NEB, E8035S) were added and incubated for additional 1 h at 4 °C. After washes, the Linc-RAM-protein complex (RNPs) was eluted with 0.1% NP-40 lysis buffer containing 20 mM maltose. The purified RNPs were separated by SDS-PAGE and stained with silver to visualize the differential protein bands. The differential bands were cut and subjected to mass spectrometry analysis.

### Western blot analysis

Skeletal muscle tissues were homogenized and lysed on ice in lysis buffer (50 mM Tris, pH 7.5, 150 mM NaCl, 0.5% Nonidet P-40, and protease inhibitor cocktail). Total proteins from skeletal muscle or the purified recombinant GST-PYGM protein were resolved by SDS-PAGE and immunoblotted using primary antibodies against PYGM (Ab81901, Abcam) overnight at 4 °C. After being washed with Tris-buffered saline containing 0.1% Tween-20 (TBST) for 30 min, the membranes were incubated with horseradish peroxidase-conjugated secondary antibodies (Zhongshanjinqiao Corporation) for 1 h at room temperature, and then washed with TBST for 30 min. The membranes were then incubated for 1 min at room temperature in Detection Solution (Thermo Scientific), and exposed to X-ray film.

### Immunofluorescent staining

C2C12 cells (2 × 10^4^ cells per cm^2^ in growth medium) were seeded in standard plastic 12-well culture plates. After the cells reached 70–80% confluence, the medium was changed to DM, and the cells were cultured for 24 h or 48 h. The cells were then fixed with 4% formaldehyde, washed with PBS, permeabilized with 0.1% Triton X-100 at room temperature, blocked with 3% bovine serum albumin for 10 min, and incubated with primary antibodies (anti-F5D diluted 1:200 or anti-MF20 diluted 1:300) for 1 h. The cells were subsequently incubated with fluorescein isothiocyanate-conjugated anti-mouse secondary antibodies (Zhongshanjinqiao Corporation) for 30 min at room temperature. MyoG (F5D, DSHB) and MHC (MF20, DSHB) staining were imaged with an Olympus IX71 fluorescence microscope (WHN × /1022; Olympus America, Inc.) equipped with the DP2-BSW software (Olympus America, Inc.). Ten representative views were taken for each sample in 12-well plates. To calculate the number of MyoG^+^ cells, the MyoG and DAPI signals were overlaid using the IPP program (Olympus America, Inc.). The merged nuclei were characterized as MyoG^+^ cells. For measurement of the fusion index, the total number of nuclei in each field of view and the total number of nuclei in multinucleated myotubes were counted using the ImageJ software (Bethesda), and the fusion index was calculated as the ratio of these two numbers.

### Real-time RT-qPCR analysis

Total RNA was extracted from cells using the TRIzol reagent (Invitrogen, Grand Island, NY, USA) and reverse-transcribed (RT) using RevertAid reverse transcriptase (Thermo Scientific). For measuring the mRNA levels of *MyoG* and *MHC*, quantitative PCR (qPCR) analyses were performed with the SsoFast EvaGreen supermix (Bio-Rad, 1,725,201). *GAPDH* was used as an internal control. All primers are presented in Supplementary Table 2.

### Nuclear–cytoplasmic fractionation

Cells were washed twice with ice-cold PBS then lysed in ice-cold PBS/0.1% NP-40 containing a protease inhibitor cocktail (Calbiochem) and ribonucleoside–vanadyl complex (10 mM; New England BioLabs). After a brief centrifugation, the supernatant was collected as the cytoplasmic fraction. The remaining pellet was subjected to additional washing and then considered the nuclear fraction. The pellet was extracted with cold nuclear lysis buffer (50 mM Tris-HCl pH 8.0, 500 mM NaCl, 1.5 mM MgCl_2_, 0.5% NP-40, 2 mM vanadyl–ribonucleoside complex). The suspension was centrifuged at 16,360×g for 20 min. The resulting supernatant was collected as the soluble nuclear fraction and the final pellet was collected as the insoluble chromatin-associated nuclear fraction.

### RNA immunoprecipitation (RIP)

Cells (2 million cells/mL) were treated with 0.3% formaldehyde in medium for 10 min at 37 °C, mixed with 1.25 M glycine dissolved in PBS to a final concentration of 0.125 M, and incubated for 5 min at room temperature. The cells were then washed twice in cold PBS and pelleted. The pellet was resuspended in 1 ml of RIPA buffer (50 mM Tris, pH 7.4, 150 mM NaCl, 1 mM EDTA, 0.1% SDS, 1% NP-40, 0.5% sodium deoxycholate, 0.5 mM DTT, and 1 mM PMSF/cocktail) and incubated on ice with frequent vortexing for 30 min, and the lysate was obtained by centrifugation at 13,000 RPM for 10 min. Antibodies (anti-Ab81901, Abcam; anti-MyoD, sc760, Santa Cruz) were added and the samples were incubated for 4 h at 4 °C and washed twice in RIPA buffer, four times in 1 M RIPA buffer (50 mM Tris, pH 7.4, 1 M NaCl, 1 mM EDTA, 0.1% SDS, 1% NP-40, and 0.5% sodium deoxycholate), and twice in RIPA buffer, all in Handee spin columns (Pierce). The beads were resuspended in RIPA buffer and treated with proteinase K at 45 °C for 45 min. RNA samples were extracted with 1 ml TRIzol, and co-precipitated RNAs were purified with an RNeasy Mini Kit (QIAGEN) and detected by RT-qPCR.

### RNA electrophoretic mobility shift assay (EMSA)

The biotin-labeled RNA probe was generated by in vitro transcription with T7 RNA polymerase (Fermentas) and biotin-UTP (Ambion). The DNA template was digested with DNase I (Promega), and the RNA probe was purified by extraction with TRIzol reagent (Ambion). The labeled RNA probe was incubated with appropriate amounts of recombinant proteins in binding buffer (10 mM Tris, pH 7.5, 1 mM EDTA, 0.1 M KCl, 0.1 mM DTT, 5% v/v glycerol, and 0.01 mg/ml BSA) with transfer RNA carrier at room temperature for 30 min. The reactions were resolved on a 5% native polyacrylamide gel and transferred to a nylon membrane (Amersham). The blot was incubated with HRP-Streptavidin (Invitrogen) and subsequently detected with ECL reagents (Thermo Scientific).

### PYGM overexpression and knockdown

The mouse *PYGM* cDNA was amplified from mouse skeletal muscle cDNA by RT-PCR and then cloned into the pcDNA 3.0 expression vector (pcDNA 3.0-*PYGM*). To overexpress PYGM, C2C12 cells were transfected with 1.6 μg pcDNA 3.0-*PYGM* plasmids per 12-plate well, using the FuGene HD transfection reagent (Roche, Basel, Switzerland). For *PYGM* knockdown, siRNAs against PYGM were designed and synthesis by Shanghai Sangon. Forward: CCGCACACAGCAGCAUUACUACGAA; Reverse: UUCGUAGUAAUGCUGCUGUGUGCGG. C2C12 were transfected with annealed siRNA, and induced for differentiation for 24 h or 48 h, respectively.

### Generation of CRISPR/Cas9-mediated *PYGM* knockout cells

The guide RNAs (sgRNAs) targeting *PYGM* gene were designed based on program developed by Feng Zhang (http://crispr.mit.edu/). Three sgRNAs were selected (Supplementary Table 2) and cloned into the pX458 vector encoding Cas9 and EGFP protein. The C2C12 cells cultured in growth medium were transfected with the pX458-sgRNA plasmids. The EGFP positive cells were sorted 48 h after transfection by flow cytometry analysis (Moflo-XDP, Beckman-Coulter) and directly seeded in 24-well plate for positive clone screening. Each clone was genotyped by PCR with two pairs of surveyor primers (Supplementary Table 2) and the PCR products were confirmed by sequencing.

### PYGM activity assay

Cells were washed twice with cold PBS and resuspended in 500 μl of TES buffer (20 mM Tris, pH 7.4, 1 mM EDTA, 225 mM sucrose, 2.5 mM DTT, 0.1 mM PMSF, 1 g/ml leupeptin, and 1 g/ml aprotinin). The samples were sonicated and centrifuged at 13,500 rpm for 10 min at 4 °C. For measurement of PYGM activity, total protein (100 μg) and 300 μl of assay buffer (50 mM KH_2_PO_4_, pH 7.5, 10 mM MgCl_2_, 5 mM EDTA pH 8, 0.5 mM NADP, 1.5 U/ml glucose-6-phosphate dehydrogenase, 1 U/ml phosphoglucomutase, and 0.1 mg/ml glycogen (all from Sigma-Aldrich)) were used. Assay buffer containing 300 μl of TES without NADP, glycogen, phosphoglucomutase, and glucose-6-phosphate dehydrogenase was added to 100 μg of total protein as a blank control. The metabolic activity assay was carried out by incubating the mixture at 37 °C for 20 min. The reaction was stopped by placing the samples on ice, and sample absorbance was detected at 340 nm in a spectrophotometer. The amount of NADPH formed was determined using a standard curve generated using known NADPH concentrations (Sigma-Aldrich).

### Statistical analysis

The results are presented as means ± SE. The statistical analyses were performed with Student *t*-tests. A *p*-value < 0.05 was considered to represent a statistically significant difference.

## Supplementary Information

**Additional file 1: Fig. S1.** Induction and purification of recombinant GST-PYGM protein. **A** Representative image of a Coomassie brilliant blue-stained SDS-PAGE gel showing the induction and purification of recombinant GST-PYGM protein using the batch method and a gravity-flow column. M: protein marker. S: soluble fraction. P: insoluble pellet. **B** Western blotting analysis verifying the identity of the recombinant GST-PYGM protein. Total proteins from skeletal muscle tissues were used as a positive control.

**Additional file 2: Fig. S2.** Schematic diagram showing regulatory sites for the enzymatic activity of PYGM. **A** Linear schematic diagram showing the relative positions of the regulated sites in PYGM. **B** Conformation of a monomer subunit of PYGM.

**Additional file 3: Supplementary Table 1.** Data from mass spectrum analysis.

**Additional file 4: Supplementary Table 2.** All primers and sgRNA sequences used in the study.

## Data Availability

All data generated or analyzed in the present study are included in this published article and the supplementary material. Requests for materials should be addressed to the corresponding author.

## Abbreviations

ncRNAs: noncoding RNAs
lncRNAs: long non-coding RNAs
Linc-RAM: linc-RNA activator of myogenesis
PYGM: glycogen phosphorylase
KO: knockout
RBPs: RNA-binding proteins
Linc-MD1: long intergenic noncoding for muscle differentiation
MAML1: mastermind-like transcriptional coactivator–1
MEF2C: myocyte enhance factor 2C
ceRNAs: competing endogenous RNAs
CHRF: cardiac hypertrophy related factor
MALAT1: metastasis-associated lung adenocarcinoma transcript
SMD: Staufen1-mediated mRNA decay
SINE: short interspersed element
IMP2: IGF2- mRNA-binding protein 2
MS2-MBP: bacteriophage MS2 coat protein and maltose-binding protein
