# Supplementary figures and images for: Linc-RAM promotes muscle cell differentiation via regulating glycogen phosphorylase activity
Source: Cell Regen. 2022 Mar 7;11:8. doi: 10.1186/s13619-022-00109-8 (PMC8901937; doi:10.1186/s13619-022-00109-8)

Figure S1

A

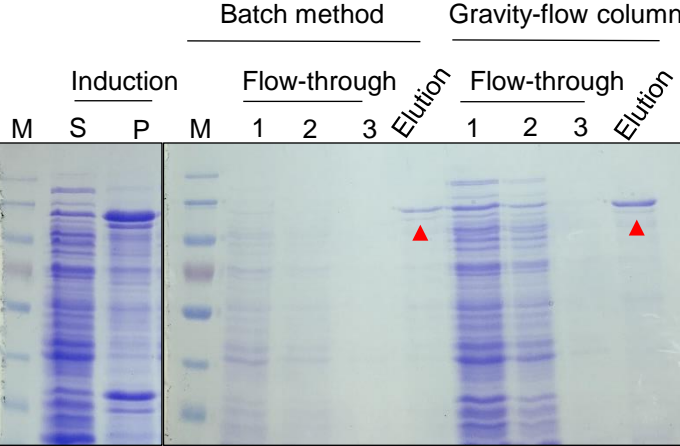

B

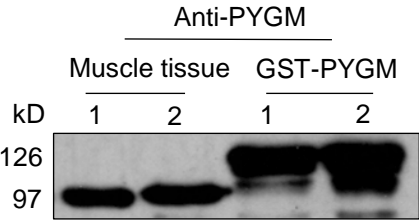

Supplement: Supplementary file 1 — Additional file 1: Fig. S1. Induction and purification of recombinant GST-PYGM protein. A Representative image of a Coomassie brilliant blue-stained SDS-PAGE gel showing the induction and purification of recombinant GST-PYGM protein using the batch method and a gravity-flow column. M: protein marker. S: soluble fraction. P: insoluble pellet. B Western blotting analysis verifying the identity of the recombinant GST-PYGM protein. Total proteins from skeletal muscle tissues were used as a positive control. [file 13619_2022_109_MOESM1_ESM.pdf]

Figure S2

A

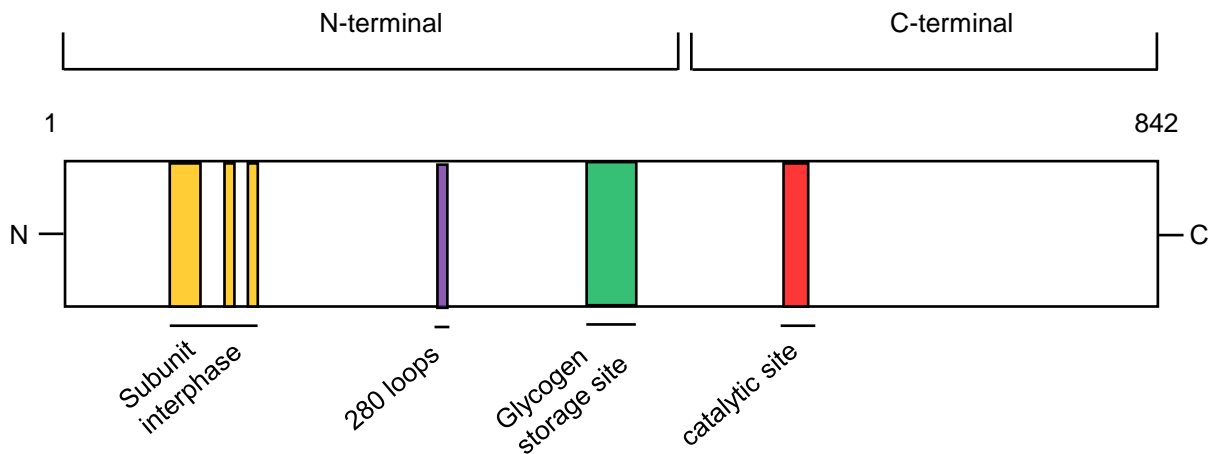

B

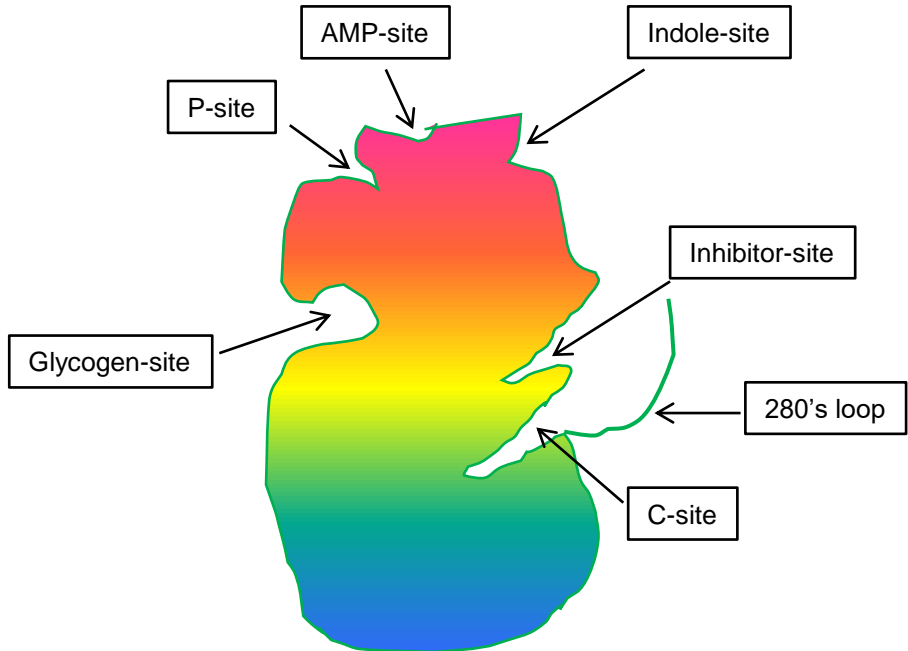

Supplement: Supplementary file 2 — Additional file 2: Fig. S2. Schematic diagram showing regulatory sites for the enzymatic activity of PYGM. A Linear schematic diagram showing the relative positions of the regulated sites in PYGM. B Conformation of a monomer subunit of PYGM. [file 13619_2022_109_MOESM2_ESM.pdf]
